# Brief Form of the Affective Neuroscience Personality Scales: Preliminary Psychometric Properties in a European Portuguese Community Sample

**DOI:** 10.1177/00332941231202016

**Published:** 2023-09-21

**Authors:** Bruno Faustino, Isabel Fonseca, Jorge Oliveira

**Affiliations:** HEI-Lab: Digital Human-Environment Interaction Labs, 70887 Lusófona University, Portugal; 37809 Faculdade de Psicologia da Universidade de Lisboa, Lisboa, Portugal; 37809 Faculdade de Psicologia da Universidade de Lisboa, Lisboa, Portugal; HEI-Lab: Digital Human-Environment Interaction Labs, 70887 Lusófona University, Portugal

**Keywords:** Brief affective neuroscience personality scales, affective neuroscience, confirmatory factor analysis, symptomatology

## Abstract

Personality theory based on affective neuroscience research suggests the presence of seven affective neurobiological systems. These dimensions have been identified using psychometric instruments such as the Affective Neuroscience Personality Scales (ANPS) and its brief version known as the Brief Affective Neuroscience Personality Scales (BANPS). Despite being a psychometric valid instrument, the BANPS was not adapted to European Portuguese. This study describes a psychometric analysis of the BANPS in a Portuguese population sample. A European Portuguese-speaking sample was recruited (*N* = 355, Mage = 27.31, DP = 12.75). Confirmatory factor analysis (CFA) was used to confirm the BANPS factorial structure. Pearson correlations were used to explore convergent validity with self-reports for psychological distress and psychopathology. Six factor model was confirmed with adequate goodness-of-fit indices (χ2(449) = 808,9841, TLI = .90, CFI = .92, RMSEA = .048 (.042–.053). Non-agreeable affective systems (anger, fear, distress), correlated positively with psychological distress and symptomology, while play and care systems correlated negatively. Seek and care subscales showed the weakest psychometric properties. The original factor structure was confirmed, suggesting the BANPS may be a valid measure to assess affective behavioral traits in the Portuguese population. Further studies in clinical populations may improve the psychometric data of the BANPS.

## Introduction

Affective neuroscience research based on animal models (Davis & Panksepp, 2018; Panksepp, 1998; Panksepp & Biven, 2012), suggests the presence of seven differentiated affective systems (anger, fear, distress, play, sex, seek and care systems) in the brain, which lie at the core of human affective behaviors (Davis & Montag, 2019; Panksepp, 1998). These systems have been studied in prior research that contributed to defining the associated behaviors. More specifically, the *anger system* is related to behavioral aggressiveness, which may be related to fighting responses (Panksepp, 1998), whereas the activation of the *fear system* is related to flight/freeze behavior such as worry, anxiety, or preoccupation (Panksepp et al., 2011). The *distress system* is triggered by the breakup of social bonds, loss or separation distress, which tends to be associated with grief feelings (Coenen et al., 2011; Panksepp et al., 2011). The *play system* involves nonharmful mental human interactions that facilitate the development of social bonds and adaptive affective experiences (Burgdorf et al., 2011; Siviy & Panksepp, 2011). The activation of the *seek system* relates to manifestations of reward-induced behaviors, namely toward pleasurable, gratification, and satisfactory experiences (Alcaro & Panksepp, 2011; Panksepp, 1998). The *care system* relates to behavioral tendencies towards social bonding, nurturance, and loving behaviors which may also include affective and pain regulation (Panksepp, 1998). Finally, the *sexual system* relates to behaviors of seek sexual patterns and sexual gratification (Panksepp, 1998).

The Brief Affective Neuroscience Personality Scales (BANPS, Barrett et al., 2013), was developed to improve the Affective Neuroscience Personality Scales (ANPS, Davis et al., 2003). The ANPS was developed to be a self-report measure by assessing overt behaviors and experiences related to the previous six neurobiological affective systems (Davis & Panksepp, 2004). The *sexual system* was not included due to the socially desirable responses (Davis et al., 2003). A *spirituality subscale* was included in ANPS despite not having a biological rationale, but not in the BANPS. Several studies were conducted suggesting correlations between ANPS with the Five-factor model (FFM), Cloninger’s Temperament, Character Inventory (TCI; Cloninger et al., 1993) and Temperament Evaluation of Memphis, Pisa, Paris, and San Diego Autoquestionnaire (TEMPS-A; Akiskal, et al., 2005) – for more detail, see Barrett and colleagues (2013). Despite this preliminary evidence of the associations with ANPS and other human personality instruments, some limitations were identified by Barrett and colleagues (2013). The ANPS was too long (112 items) and took more than 15 minutes to complete, whereas in research settings (with complex research paradigms - several scales and/or experimental settings) a short scale is preferable. Scores in *distress* as *fear systems* were highly correlated, suggesting that they are not measuring distinct constructs. Some items were ambiguous and/or poorly worded. Finally, the factorial structure of the ANPS was challenging to interpret. Therefore, the BANPS was developed to address these issues (Barrett et al., 2013).

The BANPS (Barrett et al., 2013), was developed through several studies. Study 1 was concerned with the development of BANPS scales, shortening the ANPS while maintaining or improving its psychometric properties. This study followed several steps, such as conceptual identification of problematic items, and empirical identification of good items based either on internal reliability and/or factor analysis. High correlations (*r* = .73–.92) between ANPS and BANPS subscales were found, suggesting a large, shared variance between these instruments (Barrett et al., 2013). Study 2 was concerned with the exploration of the factor structure and the psychometric properties of the BANPS in a different sample. In the first confirmatory factor analysis (CFA), the model showed adequate indexes with a combination of SRMSR and root-mean-square error of approximation (RMSEA) fit indices, χ2(480) = 1970, SRMSR = .0645, RMSEA = .0649 (.0619 −.0679), while multi-group CFA confirmed that the factorial structure of the BANPS was similar to Study 1 and (χ2(21) = 32.1602, *p* = .0564, RMSEA = .03 (0−.0498)). Moreover, to replicate a higher-order structure of the BANPS, with second level-factors, a CFA was estimated with only one specified factor loading on each factor. The model was not statistically significant χ2(8) = 47, SRMSR = .0544, RMSEA = .0818 (.0602–.1050). In the following model, the authors set the cross-loadings (e.g., play to cross-load onto negative affect and anger and sadness to cross-load onto positive affect), which improved the goodness-to-fit indexes to excellent (χ2(3) = 38, *p* < .01, SRMSR = .0189, RMSEA = .0348 (0−.0685)) (Barrett et al., 2013). Moreover, the correlations between BANPS and other measures were generally consistent. For instance, *play*, *seeking, care,* and *positive affect* correlated negatively with the effect neutral low arousal AVI subscale (Affect Valuation Index), while *fear*, *anger*, and *distress* correlated negatively with low positive arousal AVI subscale (see Barrett et al., 2013; for more detail). In Study 3, the BANPS was applied to a different sample, using a 5-point rating scale (the previous version has a 4-point rating scale), and then it was tested if the modified version would enhance the reliability of the BANPS. Also, several correlations were performed with previous measures to explore and validate if the new scale would have the same psychometric properties as the previous 4-point rating scale. Significant gender differences were found in *play*, *seek*, *care*, *fear*, *anger*, and *distress* scores where females score higher than males in all subscales. Small differences in correlational patterns were found between Study 2 and Study 3, where the vast majority were related to the degree of intensity of the correlations (Barrett et al., 2013).

In a parallel study, Pingault and colleagues (2012) also developed a short version of the Affective Neuroscience Personality Scales, which was designated as ANPS-S. This study followed some of the steps of Barrett and colleagues (2013) study considering the application of the ANPS-S to different samples (Frech and Canadian). Inter-item correlations ranged from .27 (play) to .39 (anger) in the French sample and .22 (care) to .36 (anger) in the Canadian sample. Cronbach’s alphas ranged from .61 (seek) to.79 (anger) in the French sample and .60 (play) to .71 (distress) in the Canadian sample. Finally, both samples revealed strong correlations between ANPS and ANPS-S (.81–.92).

To the best of our knowledge, ANPS-S and BANPS have not been studied together, which may be a necessary step to explore the psychometric properties of both instruments in the same study. However, for this study to be carried out, both versions must be validated and established in the same language with different samples, which is not the case for the European Portuguese population. The ANP-S and the BANPS are not validated for Portuguese-European readers and this paper focuses only on the preliminary psychometric properties of the BANPS (Barrett et al., 2013). Therefore, the study of these two instruments together is beyond the scope of this paper.

Moreover, cultural adaptation remains a fundamental hallmark of psychological testing, along with the psychometric study of the assessment measures (Urbina, 2004). According to the American Psychological Association (American Psychiatric Association, 2013), psychological assessment is based on well-established and validated instruments that allow clinicians and researchers to assess different psychological constructs. Within the dissemination of psychological instruments, the need for validation and cultural adaptations has increased in recent years to better match individuals’ characteristics. A validated psychological instrument is adapted to the cultural specifications of each population based on culture, linguistic expressions and symbolic concepts (Urbina, 2004). At the time of the execution of the present study, the BANPS has been translated into Persian (Amiri & Marzabadi, 2018) and Brazilian-Portuguese (Gurfinkel et al., 2019). However, there is a significant difference in symbolism, social concepts, semantics and contextual background between these two forms of the Portuguese language. In this sense, cultural differences and the lack of psychometric properties limit the application of the BANPS to European Portuguese speakers and a specific BANPS study in a European Portuguese sample is a requirement for psychological assessment standards (American Psychiatric Association, 2013). Although the ANPS has been translated into different languages, such as French (Pahlavan, et al., 2008), Spanish (Abella et al., 2011), Turkish (Özkarar-Gradwohl et al., 2014), Italian (Pascazio et al., 2015), Serbian (Montag et al., 2019) and German (Reuter, et al., 2017), a version of the BANPS was not available to European Portuguese psychologists and neuroscientists. Therefore, the present study aims to adapt and study the BANPS in terms of psychometric properties to European Portuguese.

## Methods

### Sample

The sample consists of 355 participants where 73 were men (20.6%) and 282 were women (79.42%). The age of the men ranged between 18 and 66 years (Mean = 28.7; SD = 14.65) whereas the age of the women ranged between 18 and 64 years (mean = 24.23; SD = 10.95). Educational level frequencies were 2 (.6%) with basic 9 years of study, 256 (72.1%) with 12th grade, 75 (21.1%) with a bachelor’s degree, and 23 (6.2%) with a master’s or doctoral degree. Relationship status were 286 (80.6%) as single, 48 (13.5%) married, 7 (2.0%) in fact union and 14 (1.4%) divorced – see Table 1.

**Table 1.** Sociodemographic Variables of the Two Samples Understudy.

		Sample	
Sample size		355	
Mean age (SD)		25.15	(11.9)
Gender (Freq - %)	Female	282	79.4
	Male	73	20.6
Nationality (Freq - %)	Portuguese	341	96.6
	Brazilian	8	2.3
	Mozambican	1	.3
	Italian	1	.3
	Costa-Rica	1	.3
	Dominican Republic	1	.3
	Ukraine	1	.3
	French	1	.3
Education (Freq - %)	9° year	2	.6
	12° year	256	72.1
	Bachelor’s degree	75	21.1
	Master´s degree	17	4.8
	Doctoral degree	5	1.4
Relationship status	Single	286	80.6
	Married	48	13.5
	Fact union	7	2.0
	Divorced	14	3.9
Psychotherapy process	Yes	86	24.2
	No	269	75.8

### Instruments

#### Brief Form of the Affective Neuroscience Personality Scales

##### Adaptation of the BANPS to European Portuguese

All the processes related to translation and cultural adaptation were aligned with Brislin’s (1980), guidelines. The BANPS was translated to European Portuguese with authorization from the original author Dr. Frederick S. Barrettt, PhD

Stage 1
First, the BANPS was translated into Portuguese by a bilingual speaker (English Portuguese).

Stage 2
Second, a back-translation was carried out by another independent bilingual speaker. Two versions were then compared, and semantic changes were conducted to match the conceptual clarity of the original items.

Stage 3
Final draft/version was revised by the author.

Stage 4
Final version was completed by 10 individuals without previous knowledge about the BANPS, to verify conceptual and linguistic clarity.

Stage 5
Without changes from the previous step, the final version of the BANPS was available to participants.

The Brief Form of the Affective Neuroscience Personality Scales (BANPS, Barrett et al., 2013; adapted to Portuguese by Faustino et al., 2022), is a self-report measure with 33 items that assess 6 affective neurobiological systems (*anger, fear, distress, play, seek* and *care systems*) underlying emotional behaviors. It has a 5-point Likert scale (1 = totally disagree to 5 = totally agree) and higher values reveal a higher frequency of affective congruent behaviors with a discrete neurobiological system. Some item examples may be given. The Seek subscale has item 3 “I am usually not highly curious”; the Fear subscale has item 15 “I often worry about the future”. Cronbach’s alpha values in the original study were the following: *anger* (α = .69), *fear* (α = .66), *distress* (α = .56), *play* (α = .68), *seeking* (α = .55) and *care* (α = .56). For the present study, see Table 2 for internal reliability.

**Table 2.** Descriptive Statistics for BANPS Scales, BSI-53 General Index and Subscales, and Psychological Well-Being and Distress Scales.

	N	α	Amp	Min	Max	Mean	SD	As	KS
Disagreeable Affect (BANPS)	355	.89	2.76	1.86	4.62	3.39	.56	−.27	−.27
Agreeable Affect (BANPS)	355	.86	2.69	2.03	4.72	3.80	.48	−.56	.30
Distress system (BANPS)	355	.88	3.83	1.17	5.00	3.30	.88	−.17	−.67
Anger system (BANPS)	355	.73	3.83	1.17	5.00	2.82	.75	.19	−.70
Fear system (BANPS)	355	.71	3.00	2.00	5.00	4.03	.61	−.63	.41
Play system (BANPS)	355	.85	4.00	1.00	5.00	3.79	.72	−.71	.45
Care system (BANPS)	355	.75	3.75	1.25	5.00	3.91	.78	−.66	.15
Seek system (BANPS)	355	.69	3.17	1.83	5.00	3.71	.57	−.39	.27
Psychological distress (MHI-5)	355	.78	5.00	.00	5.00	2.98	.63	.20	1.17
Psychological well-being (MHI-5)	355	.72	5.00	.00	5.50	3.33	.49	−.90	7.22
General symptoms index (BSI-53)	324	.97	3.64	.04	3.68	1.25	.69	.36	−.33
Somatization (BSI-53)	324	.85	4.00	.00	4.00	.89	.78	.92	.61
Obsessive Compulsive (BSI-53)	324	.81	3.83	.00	3.83	1.65	.83	.17	−.57
Interpersonal sensitivity (BSI-53)	324	.82	3.75	.00	3.75	1.44	.92	.28	−.59
Depression (BSI-53)	324	.89	4.00	.00	4.00	1.36	.92	.46	−.46
Anxiety (BSI-53)	324	.85	3.83	.00	3.83	1.34	.85	.52	−.45
Hostility (BSI-53)	324	.78	3.60	.00	3.60	.95	.71	1.02	1.05
Phobic anxiety (BSI-53)	324	.84	3.80	.00	3.80	1.01	.92	.90	.16
Paranoid ideation (BSI-53)	324	.73	3.80	.00	3.80	1.36	.74	.27	−.23
Psychoticism (BSI-53)	324	.75	3.60	.00	3.60	1.20	.82	.49	−.33

*Note*. ST: α = Cronbach alpha; Standard-Deviation; Amp = Amplitude; Min = Minimum; Max = Maximum; AS = Asymmetry; KS = Kurtosis.

### Mental Health Index

The Mental Health Index (MHI-5, translated and adapted to Portuguese by Ribeiro, 2011), is a self-report instrument that aims to assess general psychological distress and well-being. It has 5 items on a rating scale of six points (1 = all the time to 6 = none of the time). Some item examples may be given. The psychological well-being subscale has item 1 “During the past month, how much of the time were you a happy person?”; the psychological distress subscale has the item “How much of the time, during the past month, have you been a very nervous person?”. In the Portuguese adaptation study (*N* = 609), internal consistency was strong in both subscales (α = .95 for psychological distress and α = .91 for psychological well-being (Ribeiro, 2011). Higher values mean a higher presence of the scale construct. See Cronbach’s alphas in Table 2.

### Brief Symptoms Inventory

The Brief Symptom Inventory (BSI-53; Derogatis, 1993, translated and adapted to Portuguese by Canavarro, 1999) is a self-report measure that aims to assess several psychopathological symptoms. It has 53 items on seven subscales (e.g., anxiety, depression, psychoticism, hostility), with a five-point rating scale (0 = never to 4 = many times). Some examples may be given. The somatization subscale has item 7 “To have pain in the chest or in the heart”; the depression subscale has item 18 “To have feelings of guilt”. In the Portuguese adaptation study (*N* = 404), internal consistency ranged from weak in the psychoticism subscale (α = .62) to strong in the somatization subscale (α = .80) Canavarro (1999). Higher values mean a higher presence of the subscale construct. Cronbach’s alphas in Table 2.

### Procedures

Participants were recruited from a course in psychology from Faculty of Psychology of the Lisbon University and had 3 days to complete the online questionnaires. They were hosted on the Qualtrics platform. For participation, individuals received one credit for bonification. All responses were mandatory, and this information was clearly stated in the informed consent at the beginning of the study. It was also explicit that they could withdraw at any given moment. Participants also had to answer a brief sociodemographic questionnaire with some closed questions (yes or no) about self-report diagnosis (e.g., “Do you have a diagnosed neurocognitive disorder?”). Inclusion criteria were the following: being over 18 years old, speaking Portuguese as a native language (or speaking for more than 5 years), and not having a neurodevelopmental and/or neurodegenerative disorder. Four procedures were used to explore data validity: (1) time completion was checked to avoid random and fast responses (below 15 minutes were excluded and higher than 1 hour); (2) responses with all 1 or all 5 were excluded; (3) duplicate IPs were checked and excluded and (4) only the participations that fulfilled all the instruments were considered valid (Aust et al., 2013). This study was approved by the ethics committee and deontology of the Faculty of Psychology of Lisbon University and CEDIC from the School of Psychology and Life Sciences from Lusofona University. This study was not preregistered. All study procedures were aligned with Helsinki Convention.

### Statistical Analysis

The basic description of the sample was done with descriptive statistics. Confirmatory Factor Analysis (CFA) with maximum likelihood estimation was used to explore the factorial structure of the BANPS. Skewness and Kurtosis were used to explore the normality of the variables. The criteria for the CFA model were Chi-2 (χ2) with a ratio <5 as acceptable, as well as Comparative Fit Index (CFI) and a Tucker–Lewis index (TLI), with a cut-off ≥.90 as acceptable (Meyers et al., 2016). Root Mean Square Error of Approximation (RMSEA) with a value <.08 was considered acceptable (Meyers et al., 2016). According to Kline (2016), a sample size higher than 200 may be considered acceptable for CFA. Cronbach’s alpha, higher than α ≥ .70 was considered acceptable for internal reliability (Kline, 1999). Correlational analysis was performed with Pearson moment-to-moment statistics. Finally, a t-test for independent sub-samples was used to explore mean differences between a high symptom subsample and a lower symptoms subsample. All analyses were performed in 25 SPSS and AMOS software versions.

## Results

Table 2 shows Cronbach alphas, amplitude, minimum, maximum, means, standard deviations, skewness and kurtosis of the BANPS scales, BSI general index abs subscales, and psychological well-being and distress scales.

### Confirmatory Factor Analysis

**Figure 1.**
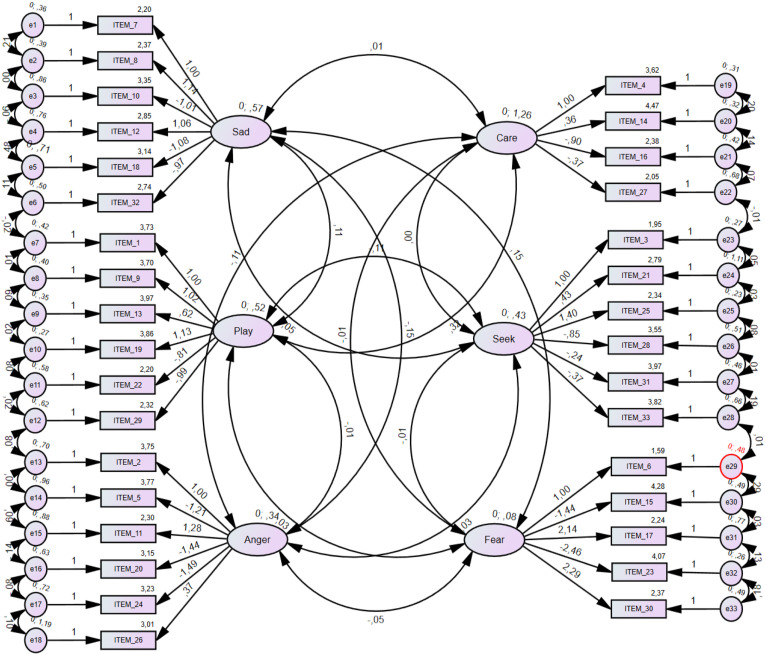
Confirmatory factor analysis for the brief affective neuroscience personality scales.

The data from the total sample (*N* = 355) was used in the CFA analysis as it provided a homogenous community sample. Only a six-model dimension was tested to match the original study by Barrett and colleagues (2013). In this sense, results showed the following indices: χ2(449) = 808,9841, TLI = .90, CFI = .92, RMSEA = .048 (.042–.053) – see Figure 1. These results adequately fit the data (Hu & Bentler, 1999). According to the CFA, scales were computed with several reversed items to match adequate levels of internal consistency (Table 2).

### Correlational Analysis

Based on the original study, two higher-order factors were computed: *Disagreeable Affect* with scales of *distress*, *anger*, and *fear systems* and *Agreeable Affect* with scales of *play*, *care*, and *seeking systems*. This nomenclature was used because primary affect tends to be viewed as adaptive from a psychological change perspective. Mourning a loss is not agreeable but is necessary to overcome this emotional state. Assertive anger is necessary to maintain social boundaries, which is adaptive but tends not to be an agreeable experience. We believe that positive and negative terms to describe emotional experience and function are outdated. *Disagreeable affect*, on one hand, correlated negatively with *agreeable affect, r(353)* = −.14, *p* < .001 and with *play system, r(353)* = −.14, *p* < .05, while on the other hand correlated positively with *distress, r(353)* = .84, *p* < .001, *anger, r(353)* = .67, *p* < .001, and *fear systems, r(353)* = .75, *p* < .001. *Agreeable affect* correlated negatively with *distress, r(353)* = −.12, *p* < .05 and *anger systems, r(353)* = −.14, *p* < .001 and correlated positively with *play, r(353)* = .79, *p* < .001, *care r(353)* = .73, *p* < .001 and *seek systems, r(353)* = .51, *p* < .001 – see Table 3. Note that the highest correlations are between the scales and the respective congruent factors (e.g., fear systems and disagreeable affect), identifying a statistical dependence.

**Table 3.** Correlational Analysis for BANPS Scales.

	Disagreeable Affect	Agreeable Affect	Distress system	Anger system	Fear system	Play system	Care system	Seeking system
Disagreeable Affect	—							
Agreeable Affect	−.14^**^							
Distress system	.84^**^	−.12^*^						
Anger system	.67^**^	−.14^**^	.27^**^					
Fear system	.75^**^	−.06	.55^**^	.23^**^				
Play system	−.14^*^	.79^**^	−.17^**^	−.03	−.10			
Care system	−.07	.73^**^	.08	−.21^**^	.03	.37^**^		
Seeking system	−.09	.51^**^	−.10	−.02	−.72	.22^**^	.01	—

*Note.* * = *p*.<05; ** = *p*.<01.

Correlational patterns show that *distress*, *anger*, and *fear systems* correlated positively with *psychological distress*, GSI, and all BSI-53 subscales (*p* < .001). Only the FEAR system did not correlate with *psychological distress*, but it did correlate with *psychological well-being* (*p* < .001). *Play system* correlated negatively with *psychological distress, r(353)* = −.19, *p* < .001, GSI, *r(353)* = −.33, *p* < .05, *somatization, r(353)* = −.18, *p* < .001, *depression, r(353)* = −.14, *p* < .05, *paranoid ideation, r(353)* = −.16, *p* < .001, *psychoticism*, *r(353)* = −.13, *p* < .05. *Care system* correlated negatively with *psychological distress, r(353)* = −.11, *p* < .001 and *paranoid ideation, r(353)* = −.13, *p* < .05, and positively with *well-being, r(353)* = −.11, *p* < .05. *Seek system* correlated negatively with *interpersonal sensitivity, r(353)* = −.13, *p* < .05, and *depression, r(353)* = −.14, *p* < .05 – see Table 4.

**Table 4.** Correlational Analysis for BANPS Scales, BSI-53 General Index and Subscales, and Psychological Well-Being and Distress Scales.

		Distress system	Anger system		Fear system	Play system	Care system	Seeking system	DA	AA
Mental health Index-5										
Psychological distress	.41^**^		.13^*^	.03		−.19^**^	−.11^**^	.02	.28^**^	−.16^**^
Psychological well-being	−.01		−.03	−.16^**^		−.10	.13^*^	−.04	−.09	−.11^*^
Brief Symptomatic Inventory										
General symptoms index (BSI)	.59^**^		.27^**^	.33^**^		−.12^*^	−.07	−.07	.38^**^	−.18^**^
Somatization	.39^**^		.18^**^	.25^**^		−.18^**^	−.09	−.09	.45^**^	−.05
Obsessive Compulsive	.48^**^		.18^**^	.30^**^		−.02	−.02	−.07	.47^**^	−.13^*^
Interpersonal sensitivity	.52^**^		.19^**^	.30^**^		−.10	−.05	−.13^*^	.56^**^	−.16^**^
Depression	.68^**^		.22^**^	.28^**^		−.14^*^	−.06	−.14^*^	.52^**^	−.04
Anxiety	.53^**^		.20^**^	.39^**^		−.07	−.01	.01	.48^**^	−.08
Hostility	.35^**^		.54^**^	.15**		−.04	−.08	−.03	.38^**^	−.03
Phobic anxiety	.41^**^		.12^*^	.29^**^		−.05	−.01	.01	.38^**^	−.19^**^
Paranoid ideation	.39^**^		.22^**^	.21^**^		−.16^**^	−.13^*^	−.10	.52^**^	−.13^*^
Psychoticism	.58^**^		.25^**^	.30^**^		−.13^*^	−.10	−.03	.38^**^	−.18^**^

*Note*. * = p.<05; ** = p.<01. DA = Disagreeable Affect: AA = Agreeable Affect.

### T-Test for Independent for Independent Samples

Gender differences between men and women were found in *distress t(353)* = −5.03, *p* < .001), *anger t(353)* = −2.86, *p* = .004), *fear t(353)* = −5.98, *p* < .001), *seek t(353)* = 2.53, *p* < .012) and *disagreeable affect subscales t (353)* = −6.15, *p* < .001) – see Table 5. Also, BSI-53 (Portuguese version) has a cutoff value (1.7<) which is a valid empirical criterion to identify individuals with clinically significant symptomatology. Individuals with the General Symptomatic Index (GSI) higher than 1.7 tend to manifest clinically significant symptoms (Canavarro, 1999). Therefore, it is possible to split the total sample into two different subsamples, where one sample has individuals with the highest symptoms (HSS, High Symptoms Sub-sample), while the other has individuals with the lowest symptoms (LSS, Low Symptoms Sub-sample). Table 5 Shows that on one hand, mean values in the *distress t(322)* = 8.97, *p* < .001), *anger t(322)* = 4.01, *p* < .001), *fear t(322)* = 5.90, *p* < .001), and *disagreeable affect subscales t(322)* = 8.81, *p* < .001) are significantly higher in the HSS when compared to the LSS, while on the other hand, mean values in the *play subscale t(322)* = −1.67, *p* < .001), are higher in the LSS when compared to HSS.

**Table 5.** T-Test Comparisons for BANPS Subscales Between the High Symptoms Sub-Sample and Low Symptoms Sub-Sample and Between Gender.

Gender		N	Mean	SD	t	Sig	Samples	N	Mean	SD	t	Sig
Distress	Men	73	2.86	.89	−5.03	.01	HSS	87	3.95	.64	8.97	.01
	Women	282	3.42	.84			LSS	237	3.06	.84		
Anger	Men	73	2.60	.75	−2.86	.04	HSS	87	3.08	.77	4.01	.01
	Women	282	2.88	.74			LSS	237	2.71	.72		
Fear	Men	73	3.67	.63	−5.98	.01	HSS	87	4.33	.50	5.90	.01
	Women	282	4.13	.57			LSS	237	3.90	.62		
Play	Men	73	3.88	.81	1.21	.22	HSS	87	3.68	.80	−1.67	.01
	Women	282	3.77	.70			LSS	237	3.83	.71		
Care	Men	73	3.85	.82	−.74	.46	HSS	87	3.85	.87	−.82	.41
	Women	282	3.93	.77			LSS	237	3.93	.76		
Seek	Men	73	3.86	.55	2.53	.01	HSS	87	3.71	.58	−.01	.99
	Women	282	3.67	.57			LSS	237	3.71	.58		
DA	Men	73	3.04	.58	−6.15	.01	HSS	87	3.78	.41	8.81	.01
	Women	282	3.47	.53			LSS	237	3.22	.54		
AA	Men	73	3.87	.51	1.20	.22	HSS	87	3.74	.55	−1.28	.19
	Women	282	3.79	.47			LSS	237	3.82	.45		

*Note*. HSS = High Symptoms Sub-sample; LSS = Low Symptoms Sub-sample; DA = Disagreeable Affect: AA = Agreeable Affect.

## Discussion

The present work detailed a psychometric study of the Brief Form of the Affective Neuroscience Personality Scales (BANPS) in a Sample of the Portuguese Population. The overall results were satisfactory. Through a confirmatory factor analysis (CFA) it was possible to replicate the six-factor structure suggested by the original study of the BANPS (Barrett et al., 2013). The same structure was found by Pingault and colleagues (2012) in a parallel study with a short version of the Affective Neuroscience Personality Scales (ANPS) named (ANPS-S). These results suggest a consistent latent structure of the ANS despite being different versions. ANPS was developed as a self-report instrument to assess endophenotypes directly related to subcortical brain emotional systems that contribute to the generation of core aspects of human affective experiences (Panksepp, 2010). Human affective experience tends to range from agreeable to disagreeable experiences which reflect core sensations regarding different emotional valence (Davis & Panksepp, 2018). This is consistent with BANPS conceptualization and the underlying factor structure. It is noteworthy that an increase in Cronbach’s alpha values from the original BANPS, suggests an adequate interrelatedness between the items of the subscales in the present study. One likely explanation may be related to the degree to which the participants interpreted the items as more similar to each other than in the original BANPS study. According to Kline (1999), higher values of alphas suggest higher item homogeneity which may reflect this effect. In this sense, further studies should explore if these Cronbach’s alpha values are stable in the other European Portuguese samples.

Convergent validity was explored through bivariate correlations between the BANPS subscales with the convergent measures used in our study. Most of the correlational patterns were consistent with previous research findings and were aligned with theoretical predictions. Nevertheless, some results are challenging to interpret. The care and seek systems did not negatively correlate with the disagreeable affect factor, which suggests that behaviors and/or traits based on caring and seek motivational systems may be more concerned with the “motivational” aspect of the tendency, rather than the reinforcement of the avoidance of disagreeable affect. In other words, if an individual is motivated to manifest caring behaviors (e.g., nurturance, social bonding), the primary reward may be to establish and maintain these connections with others. An individual who is high on seek behaviors may also be more focused on the need to receive a reward, rather than the specific affect that stems from the reward. Moreover, *play*, *seek*, and *care systems* were positively correlated with *agreeable affect*, which is in line with the study from Montag and Davis (2018) that showed positive correlations between *care* and *seek systems* with agreeableness trait measured by the Big-Five Inventory (BFI, John et al., 2008).

The correlations between BANPS sub-scales and symptomatology were also theoretically consistent with previous findings. *Distress*, *anger*, and *fear systems* which composed the disagreeable affective factor correlated positively with all symptomatic domains of the Brief Symptomatic Inventory (BSI-53), which suggests that these core disagreeable affective systems may contribute, at least in part, to the experience of psychopathological symptomatology. Thus, Montag and Davis (2018) described that distress, anger, and fear systems are correlated with the personality trait of neuroticism and with all their facets/subscales (e.g., depressed, irritated, stressed). These results are also aligned with previous correlational patterns between ANPS and BFI, where distress, anger, and fear systems were strongly correlated with neuroticism while *seek* was correlated with openness, care with agreeableness and play with extraversion (Barrett et al., 2013; Davis et al., 2003). The *care* and *seek* subscales in the present study, by comparison to other BANPS subscales, were the ones with less significant correlations. This was not expected because all the other subscales showed adequate correlational patterns. One aspect is concerned with the internal consistency of these two scales, which was low. Another possible explanation may be that the original items of the BANPS may not capture the nature of the construct in the Portuguese population. We suggest that future studies would explore a different wording of the items in these two specific subscales. Nevertheless, despite having less significant correlations than the other subscales, their pattern was consistent with expected predictions.

Gender differences showed that women had significantly higher mean values that men in *distress*, *ange*r, *fear* and *disagreeable affect* subscales and men had higher mean values in *seek* than women. In the original study, *care*, *distress* and *fear* were higher in women, whereas *anger* was higher in men (Barrett et al., 2013). These results suggest that on one hand, this study replicated partially the original results mainly in the *disagreeable affect subscales,* and on the other hand, it revealed differences in the *seek* and *anger* subscales. Maybe this could reflect cultural differences in affective neurobiological systems between men and women. Further studies should focus on the stability of gender differences in European Portuguese-speaking samples.

Also, differences were found in the mean values of the BANPS scales in two subsamples divided by the BSI index (1.7 <). Mean values of *distress*, *anger*, and *fear systems* were significantly higher in the high symptoms subsample than the low symptoms subsample, whereas the mean value of the *play system* was significantly higher in the low symptoms subsample that the higher symptoms subsample. No significance was found for *care* and *seek* scales. One possible explanation may be that individuals do not differ in caring and seek motivational tendencies despite having clinically significant symptoms. Another possible explanation may be concerned with the number of women in the study sample, which is far superior to men. It is established in the literature that women are more prone than man to experience anxiety, depression, and distress (Leibenluft, 1999; Parker &, Brotchie, 2010).

### Clinical Considerations, Limitations Future Directions

Some clinical considerations may be noteworthy regarding affective neuroscience research and BANPS. The study of human affective neurobiological systems may contribute to a unified metatheory between neurobiology and psychology, that help clinicians and researcher to better conceptualize interactive gene and environment interplay that leads to emotional suffering and behavioral difficulties. By mapping specific underlying neurobiological systems related to affective behavioral tendencies it is possible to better conceptualize which clinical strategy is better suited for the specific individual. For instance, if an individual with an affective disorder, is high on distress and low on care, may benefit from clinical strategies focused on increasing social bonding interactions and behaviors. Also, efforts are underway to explore relationships between emotional core needs, motivational systems, and psychosocial stages with affective neurobiological systems. It has been theorized that these neural systems are the basis for higher psychological constructs, such as emotional needs, motivations, and values (Faustino, 2022) and may be related to complex neural network impairment, such as in the Default Mode Network (Faustino, 2021; Faustino et al., 2022). Attention to the applicability of affective neuroscience concepts to psychotherapy should be tackled in the future. Thus, associations between affective neuroscience and symptomatology have been recently described in the literature (Montag & Davis, 2018).

Finally, some limitations can be described. First, this study was carried out with individuals with a high level of education, which limits extrapolations to other individuals with a lower level of education. Secondly, the size of the sample does not allow for a coherent representation of the Portuguese population. Third, this study had more women than men, which may induce some response bias. Third, convergent validity can also be explored with other instruments, such as the SMS (Brasini et al., 2020). Fourth, discriminant validity was not performed and may be performed in future studies. Fifth, disagreeable and agreeable affect scales were computed based on the original BANPS study (Barrett et al., 2013). However, their factor structure was not tested in the present study. In this sense, subsequent studies may explore an SEM model to address this issue. Also, future studies should focus on enhancing the psychometric properties of the BANPS, namely by addressing the issue of *care*, *seek* subscales, and exploring the BANPS in other samples.

## Conclusions

The present work detailed a psychometric study of the Brief Form of the Affective Neuroscience Personality Scales (BANPS) in a Sample of the Portuguese population. Confirmatory and correlational analysis showed that BANPS may be a reliable and useful instrument to be used by clinicians and researchers in a variety of settings.
